# Demonstrating a link between diet, gut microbiota and brain: ^14^C radioactivity identified in the brain following gut microbial fermentation of ^14^C-radiolabeled tyrosine in a pig model

**DOI:** 10.3389/fnut.2023.1127729

**Published:** 2023-03-09

**Authors:** Margaret Murray, Christopher K. Barlow, Scott Blundell, Mark Buecking, Anne Gibbon, Bernd Goeckener, Lisa M. Kaminskas, Patricia Leitner, Sophie Selby-Pham, Andrew Sinclair, Habtewold D. Waktola, Gary Williamson, Louise E. Bennett

**Affiliations:** ^1^School of Chemistry, Monash University, Clayton, VIC, Australia; ^2^Department of Nutrition, Dietetics and Food, Monash University, Notting Hill, VIC, Australia; ^3^Monash Proteomics and Metabolomics Facility and Monash Biomedicine Discovery Institute, Monash University, Clayton, VIC, Australia; ^4^Fraunhofer Institute for Molecular Biology and Applied Ecology, Schmallenberg, Germany; ^5^Monash Animal Research Platform, Monash University, Churchill, VIC, Australia; ^6^School of Biomedical Sciences, Faculty of Medicine, The University of Queensland, Brisbane, QLD, Australia; ^7^Faculty of Health Sciences, School of Pharmacy, Institute of Health, Jimma University, Jimma, Ethiopia

**Keywords:** biodistribution, tyrosine, resistant protein, α-linolenic acid, carbon-14 radio-isotope, gut-brain axis

## Abstract

**Background:**

There is a need to better understand the relationship between the diet, the gut microbiota and mental health. Metabolites produced when the human gut microbiota metabolize amino acids may enter the bloodstream and have systemic effects. We hypothesize that fermentation of amino acids by a resistant protein-primed gut microbiota could yield potentially toxic metabolites and disturb the availability of neurotransmitter precursors to the brain. However, these mechanisms are challenging to investigate *via* typical *in vitro* and clinical methods.

**Methods:**

We developed a novel workflow using ^14^C radiolabeling to investigate complex nutrient-disease relationships. The first three steps of the workflow are reported here. α-Linolenic acid (ALA) was used as a model nutrient to confirm the efficacy of the workflow, and tyrosine (Tyr) was the test nutrient. ^14^C-Tyr was administered to male weanling pigs fed a high resistant protein diet, which primed the gut microbiota for fermenting protein. The hypotheses were; (1) that expected biodistribution of ^14^C-ALA would be observed, and (2) that radioactivity from ^14^C-Tyr, representing Tyr and other amino acids released from resistant protein following gut microbial fermentation, would be bioavailable to the brain.

**Results:**

Radioactivity from the ^14^C-ALA was detected in tissues reflecting normal utilization of this essential fatty acid. Radioactivity from the ^14^C-Tyr was detected in the brain (0.15% of original dose).

**Conclusion:**

Metabolites of gut-fermented protein and specifically amino acid precursors to neurotransmitters such as tyrosine, are potentially able to affect brain function. By extension, resistant proteins in the diet reaching the gut microbiota, also have potential to release metabolites that can potentially affect brain function. The high specificity of detection of ^14^C radioactivity demonstrates that the proposed workflow can similarly be applied to understand other key diet and health paradigms.

## 1. Introduction

Nutrition directly influences human health, with either positive or negative impacts reflecting the balance of macro- and micro-nutrients, and other factors. It is important to understand the potential impacts of specific nutrients, foods and dietary patterns on human health, in order to inform accurate and scientifically sound nutritional advice. For example, it is important that the bioactivity of components in functional foods, claiming to enhance human health (1), be fully substantiated and based on sound scientific evidence. Likewise, there is growing interest in the impacts of ultra-processed foods on human health and it is important to understand the various mechanisms through which these products influence health (2).

It is a significant challenge to track the chemical fate of nutrients in the body due to the progressive biochemical transformations of food components associated with digestion, absorption, metabolism and excretion (3, 4). Because of this, it is difficult to demonstrate the mechanisms within the human body by which specific compounds affect physiological functions and health. While *in vitro* and rodent models can provide valuable mechanistic information, these methods may fail to replicate relevant dosage, nutrient digestion, absorption and metabolism, or the potential influence of other dietary components (5). Gold standard human intervention studies, relying on sampling of blood, feces, and urine, can demonstrate effects of nutrients on a health outcome, but are limited in demonstrating full mechanistic pathways and tissues involved.

In recent years, there has been much interest in the relationship between dietary intake, the gut microbiota and mental health (6–11). However, the mechanisms that link diet, gut microbiota and mental health are challenging to investigate using the above-described methods, as this is a complex metabolic pathway. Understanding how dietary components influence the gut microbiota and the bioactivities of resulting microbial metabolites as they contribute to the “gut-brain axis” represents a key step in characterizing the relationship between nutrition and mental health (10, 11).

Metabolites produced by the human gut microbiota, including neurotransmitters derived from amino acids (such as tryptamine, dopamine and serotonin), can enter the bloodstream and have systemic effects (10, 12–15). By extension, resistant proteins produced through high-heat processing of foods that are transported and fermented in the colon, may account for elevated release of amino acids in the colon (10, 16–18). Gut microbial fermentation of amino acids could perturb amino acid metabolism and influence brain function. For example, dysbiosis of the gut microbiota can cause an increase in tryptophan metabolic pathways that produce kynurenic acid and other metabolites, and a reduction in pathways involved in serotonin synthesis (19). Such changes in tryptophan metabolism play a role in the onset and progression of depression (19).

**FIGURE 1:**
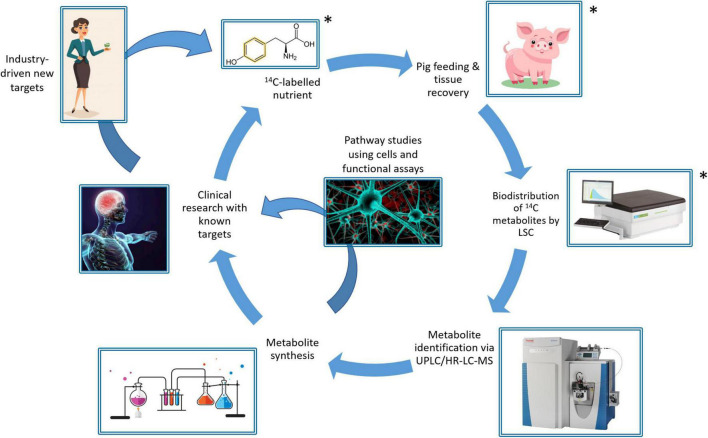
Schematic of the ^14^C-workflow for tracking the metabolic fate of bioactives/nutrients in food, showing the segments demonstrated in this study (*).

We hypothesize that gut microbial metabolism of amino acids from a high-resistant protein diet [50% *in vitro* protein digestibility compared to the standard diet (16)] could yield potentially toxic metabolites arising from fermented protein and additionally disturb the availability of neurotransmitter precursors to the brain. To investigate this, we developed a workflow (Figure 1) using ^14^C stable isotope radiolabeling of the target nutrient/bioactive compound (20). The use of ^14^C-labeling to trace the metabolic fate of nutrients is well-established, with examples including fatty acids (21), triglycerides (22), vitamins (23, 24), and toxins (25). The proposed workflow involves oral intake of the ^14^C-labeled nutrient and following a short period allowed for digestion and metabolism, subsequent collection of blood, urine, feces, organs and tissues to determine the biodistribution of ^14^C. Pigs were chosen as the pre-clinical model, since the digestive system of pigs is most closely matched to that of humans compared to other non-primate species, making them a highly clinically relevant model (26). The biodistribution of ^14^C-labeled metabolites is then characterized by measuring radioactivity in samples *via* liquid scintillation counting (LSC). Following this, specific metabolites may be identified using ultra-high performance liquid chromatography and high resolution mass spectrometry (UHPLC/HR- MS) analysis. Following identification of key metabolites, they can be chemically synthesized to closely study bioactivity in cells and functional assays. Finally, clinical research studies can investigate the impact of target nutrients on health and disease, using pre-identified biomarkers. This workflow is designed to address the challenge of demonstrating direct causal relationships between intake of dietary components and the resulting physiological outcomes, *via* the actions of bioactive compounds and their metabolites, and could be broadly applied to study diet-disease relationships.

This study aimed to (1) validate the workflow using the essential omega-3 fatty acid, α-linolenic acid (ALA), (2) investigate the biodistribution of tyrosine (Tyr), an aromatic amino acid and neurotransmitter precursor, and its metabolites following fermentation by a resistant protein-primed gut microbiota, and whether these metabolites could enter the brain, and (3) determine whether there was a difference in the metabolism of aromatic amino acid, tryptophan (Trp), between the standard diet and the resistant protein diet, demonstrating gut microbial fermentation. ALA was selected as a model compound to validate the workflow because the biodistribution of ALA is well documented (27–29), allowing for comparison between findings from the workflow and the literature to confirm that the workflow is effective at measuring biodistribution of nutrients in tissues and organs. The research reports on three steps of the overall workflow (Figure 1), including the protocol for feeding selected ^14^C-labeled nutrients to pigs, followed by tissue recovery and measuring biodistribution of total radioactivity by LSC. The results demonstrate the feasibility of the workflow for characterizing biodistribution of nutrients following both upper gut and colon fermentation-mediated absorption.

## 2. Materials and methods

### 2.1. Materials

The ^14^C-labeled nutrients: α-linolenic acid (^14^C-ALA) and L-tyrosine (^14^C-Tyr), were provided by Hartmann Analytic GmbH (Braunschweig, Germany). The ^14^C-ALA contained a single ^14^C-labeled atom at C1: [1-^14^C]-α-linolenic acid. In the tyrosine molecule, all 9 carbon atoms were ^14^C labeled: [^14^C(U)]-L-tyrosine. The radioactive compounds were formulated into capsules for consumption. The radio-labeled ALA was prepared in two size 0 capsules, each containing 1.36 mg [1-^14^C]-ALA, with an activity of 10 MBq, and 75 mg methocel (Hartmann Analytic GmbH, Germany). Identical non-labeled capsules were also prepared containing 1.36 mg ALA and 75 mg methocel (Hartmann Analytic GmbH). The ^14^C-Tyr was prepared in two capsules (size 0), each containing 0.1 mg 14C-Tyr, with an activity of 10 MBq, 64 mg citric acid and 50 mg methocel. Identical non-labeled capsules were also prepared containing 0.1 mg L-Tyrosine, 64 mg citric acid and 50 mg Methocel (Hartmann Analytic GmbH, Germany). The unlabeled and ^14^C-labeled tyrosine capsules were then enclosed in size 00 acid-resistant capsules to prevent digestion in the stomach and promote release in the colon. The strategy to deliver ^14^C-Tyr into the colon was intended to mimic amino acids contained within resistant proteins that, due to digestive resistance, are transported and released during fermentation in the large intestine (16).

Reagents for liquid scintillation counting included solvable, soluene-350, Ultima Gold, Hionic-Fluor and scintillation vials and were purchased from Perkin Elmer Pty. Ltd. (Glen Waverley, VIC, Australia). GentleMACS™ M tubes were obtained from Miltenyi Biotec Pty. Ltd. (Macquarie Park, NSW, Australia). Hydrogen peroxide was obtained from Thermo Fisher Scientific (Scoresby, VIC, Australia).

### 2.2. Experimental animals and study design

Ethical approval for the research was granted by the Monash Animal Research Platform-1 Animal Ethics Committee (reference: 17533) and the study was monitored according to the ARRIVE guidelines for animal research (30). The study protocol preceding administration of ^14^C-capsules was previously reported (16). The overall study design and details for administering the ^14^C capsules is described below. Landrace cross Large White male weanling pigs commenced the standard diet (*n* = 4) or the resistant protein diet (*n* = 4) 4 weeks prior to ^14^C-nutrient administration. Feed intake was controlled to provide 100% of energy requirements. Feed intake of individual pigs was monitored daily and water was provided *ad libitum*.

The animals that received the ALA capsules were fed the standard pig weaner diet (SF18-148 Specialty Feeds, Glen Forrest, WA) containing wheat, barley, lupines, soya meal, calcium carbonate, salt, dicalcium phosphate, lysine, and a vitamin and trace mineral premix. The animals that received the Tyr capsules were fed the high resistant protein diet (SF18-147 Specialty Feeds, Glen Forrest, WA, Australia) substituted with a protein source of high-heat-treated skim milk powder, also containing: barley, skim milk, soya meal, canola meal, calcium carbonate, salt, dicalcium phosphate, and a vitamin and trace mineral premix. The resistant protein diet was treated by autoclaving (15 h at 70°C followed by 20 min at 121°C and cooling for 2 h before vacuum packing) to model high heat/low moisture processing, and contained a high proportion of indigestible protein, that primed the colonic microbiota for protein fermentation (16).

Within each cohort, one animal received the ^14^C-labeled encapsulated nutrient and three animals received equivalent non-labeled encapsulated nutrients. Sample size of n = 1 for the ^14^C-labeled nutrient was deemed sufficient to identify the biodistribution nutrient metabolites due to the specificity of detecting radioactivity. This was also considered appropriate to reduce occupational health and safety risks associated with radioactivity, and ethically acceptable to minimize animal use.

**FIGURE 2:**
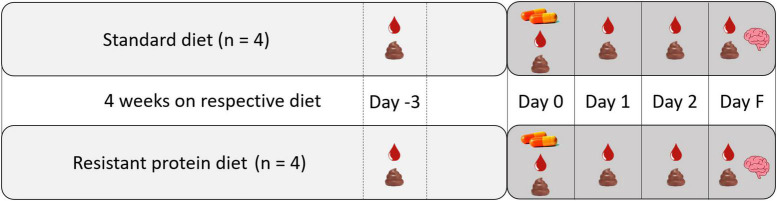
Study design showing the capsule administration and sampling protocol. Blood droplet icon indicates blood sampling, feces icon indicates excreta sampling, capsule icon indicates administration of capsules, brain icon indicates terminal sampling of tissues and organs.

On the day of capsule administration, each pig was moved into an individual metabolic cage and remained in the metabolic cage until euthanasing (3 days), and was accompanied at all times by another pig in an adjacent metabolic cage to minimize stress. Three pigs from each group were administered the unlabeled capsules, and one pig from each group was administered the ^14^C radio-labeled capsules. The capsules were administered with the morning feed (approximately 9:00 A.M.). The ALA capsules were included with the pig feed. The Tyr capsules were administered using a pill popper to ensure the acid-resistance capsules were delivered to the intestine intact. A 3-day window between capsule administration and euthanasia allowed time for metabolism and distribution of the ^14^C-nutrients into tissues and for sampling of blood and excreta to track absorption and metabolism (Figure 2).

### 2.3. Sample collection

On the day of capsule administration (day 0) and each of the following three days (day 1, day 2, and final day F), blood and combined urine and fecal samples (excreta) were collected (Figure 2). On day 0, blood samples were collected in the morning (∼11:00 a.m.) and afternoon (∼ 3:00 p.m.) for the ALA group. For the Tyr group, blood samples were only collected in the afternoon (∼ 3:00 p.m.) on day 0, due to ethical concerns of taking two blood samples in a day because of their smaller size. Blood samples were then each day thereafter (∼11:00 a.m.) until euthanasia.

Blood samples were obtained with the pigs under light anesthesia (8% sevoflurane in 100% oxygen delivered by snout mask, <1 min). Blood samples were immediately centrifuged (10 min at 1,500 × *g*, 4°C). Aliquots of plasma and blood cells were isolated and snap frozen on dry ice. Excreta samples were collected on days 0, 1, 2, and F and snap frozen on dry ice. Blood and fecal samples were also taken three days before capsule administration. All samples were stored at −80°C until analysis.

Following euthanasia by intravenous overdose of barbiturate pentobarbitone and subsequent confirmation of death, bleeding was performed to minimize blood content of tissue samples and cross-contamination of ^14^C radioactivity between blood and tissues post-mortem. Sample collection was carried out by a veterinarian with the help of trained researchers. The weight of the pig and each organ was recorded. Organ samples collected were colon, small intestine, stomach, spleen, heart, liver, kidney, lung, pancreas and brain (lobes, cerebellum and mid-brain). Tissue samples collected were skeletal muscle (quadricep and tricep) and subcutaneous abdominal adipose tissue. The contents of the stomach, small and large intestines (digesta) were also collected. All samples were snap frozen on dry ice before storage at −80°C.

### 2.4. Determination of radioactivity by liquid scintillation counting

#### 2.4.1. Determination of radioactivity in plasma and blood cells

The biodistribution ^14^C-ALA and ^14^C-Tyr were assessed by LSC. Plasma aliquots were thawed and centrifuged (3,500 rpm, 5 min). Samples from a single “cold” pig were also analyzed for background correction. Plasma supernatant (250–500 μL) was mixed with 2 mL Hionic-Fluor cocktail in 6 ml scintillation vials prior to vortex mixing and scintillation counting on a Tri-Carb liquid scintillation counter (Perkin Elmer, Waltham, MA, United States). To quantify ^14^C in blood cells, samples were thawed and vortex mixed to obtain a homogenous solution. Blood cell samples (and also tissue and excreta samples described below) were solubilized and analyzed as described previously (31) with some modification. Blood cell samples from ^14^C dosed pigs or one cold-dosed pig (100 μL) were added to 20 mL scintillation vials and mixed with 2 mL of Solvable tissue solubilizer before being placed at 60°C overnight. Samples were subsequently cooled to room temperature and bleached with 400 μL of hydrogen peroxide (30% w/v) before the addition of Ultima Gold scintillant (10 mL). Samples were left in the dark at room temperature overnight prior to storage at 4°C for 4 days to suppress chemiluminescent reactions. The samples were then analyzed by LSC in sets of twelve to minimize warming and subsequent increases in chemiluminescent background during counting. Data are reported as the percentage of the original dose of radioactivity (20 MBq) quantified in each sample, or as the level of radioactivity (Bq) per volume of sample.

#### 2.4.2. Determination of radioactivity in tissues

Tissues samples were weighed, minced into approx. 3 mm pieces and dispersed in MQ water (three-fold dilution, w/w). Tissues were homogenized in gentleMACS™ M tubes using a gentleMACS™ Dissociator (Miltenyi Biotec, Macquarie Park, NSW, Australia). An initial “screening” stage was used to determine the approximate levels of radioactivity in the samples. For this stage, a 450 μL aliquot of tissue homogenate was mixed with 2 mL Solvable in a 20 ml scintillation vial and incubated at 60°C overnight. After cooling to room temperature, 200 μL of hydrogen peroxide (30% w/v) was added to bleach the samples before Ultima Gold (10 mL) was added and the solution vortex mixed. The samples were then sequentially stored in the dark at room temperature overnight, then at 4°C for 4 days before scintillation counting as described above. Corresponding tissue samples were also analyzed from a cold pig to provide background correction.

In the second “analytical” stage, all tissue samples were analyzed in sets of 3 or 5. Homogenized tissues from ^14^C-dosed pigs were processed as described above in the “screening” stage, except that additional steps were taken to correct for any reductions in radioactivity counting efficacy that occurs in some tissue samples. Tissue homogenate from the pig receiving ^14^C-ALA (100 to 450 μL, depending on radioactive content) was aliquoted in triplicate into 20 mL scintillation vials. Tissue homogenate from the pig receiving ^14^C-Tyr (50 to 450 μL, depending on radioactive content) was weighted into 5 × 20 ml scintillation vials. From the P2 set, 3 vials were solubilized and analyzed to determine ^14^C content, while the remaining 2 vials were spiked with remaining pooled plasma from ^14^C dosed pigs at an appropriate volume to provide approximately the same ^14^C activity as the aliquoted tissue samples. Tissues were then solubilized in Solvable and treated as described above for whole blood prior to scintillation counting. The ^14^C content of tissues on a per mass and per organ basis were then analyzed (accounting for counting efficiency in each organ) as described previously (31).

#### 2.4.3. Determination of radioactivity in excreta and intestinal contents

Excreta and intestinal contents (colon digesta, stomach digesta and small intestinal digesta) were each aliquoted into 20 ml vials and prepared into a homogenous slurry by mixing in MilliQ water (three-fold dilution, w/w). An initial “screening” stage was used to determine the approximate levels of radioactivity in the samples as described above.

In the second “analytical” stage, all tissue samples were analyzed in sets of 3 or 5 as described above for tissue samples. Pooled excreta and intestinal contents from ^14^C-dosed pigs were processed as described above in the “screening” stage, except that additional steps were taken to correct for any reductions in radioactivity counting efficacy that occurs in some samples as described above for tissues. Briefly, excreta or intestinal contents with the ^14^C-ALA (100 to 450 μL, depending on radioactive content) were weighed in triplicate into 20 ml scintillation vials, while samples with the ^14^C-Tyr (50 to 450 μL, depending on radioactive content) were weighted into 5 × 20 ml scintillation vials. From the ^14^C-Tyr set, 3 vials were solubilized and analyzed to determine ^14^C content, while the remaining 2 vials were spiked with remaining pooled plasma from ^14^C dosed pigs at an appropriate volume to provide approximately the same ^14^C activity as the aliquoted samples (determined during the screening stage). The samples were then processed and analyzed for radioactivity content as described above.

### 2.5. Analysis of selected metabolites in plasma and feces by LC-MS

Plasma and fecal samples taken from both diet groups at day -3 (Figure 2), were analyzed for selected metabolites of tryptophan [tryptophan (Trp), kynurenine (Kyn), tryptamine (TA)]. Tryptophan is an aromatic amino acid and neurotransmitter precursor, similar to tyrosine. This analysis was to determine whether there was a difference in the metabolites produced from aromatic amino acids between the standard diet (normal amino acid metabolism) and the resistant protein diet (gut microbial metabolism), representing disturbed amino acid metabolism.

#### 2.5.1. Plasma sample preparation

The plasma preparation was based on a previously published method by Zhu et al. (32) with slight modification. The plasma was thawed on ice before transferring 25 μL to a 1.5 mL polypropylene tube and adding 250 μL of extraction solvent (80% methanol with 0.02% formic acid and 0.5 μM 3-methyl-2-oxindole as an internal standard). The samples were agitated using a vortex mixer at 4°C before standing at −20°C for 1 h and subsequent centrifugation (20,000 × *g*, 4°C, 10 min). The supernatant was transferred to a new tube and evaporated to dryness under vacuum. The residue was then reconstituted in 100 μL of 0.1% formic acid and subjected to centrifugation (20,000 × *g* at 4°C, 10 min) before transferring 80 μL of the supernatant to a sample vial for LC-MS analysis.

#### 2.5.2. Fecal sample preparation

Fecal samples were prepared using a similar method to the plasma samples with the following modifications. The fecal samples were allowed to thaw on ice before transferring approximately 30 to 60 mg to a 2 mL polypropylene tube. The samples were weighed and 20 μL of ice-cold extraction solvent (containing 1.25 μM of internal standard) per mg of feces was added. Samples were then agitated using a vortex mixer at 4°C before standing at −20°C for an hour and subsequent centrifugation (20,000 × g, 4°C, 10 min). A total of 500 μL of the supernatant was transferred to a 1.5 mL tube and evaporated to dryness under vacuum. The sample was then reconstituted in 250 μL of 0.1% formic acid, followed by centrifugation (20,000 × *g* at 4°C, 10 min) before transferring approximately 180 μL of the supernatant into a sample vial for LC-MS analysis.

#### 2.5.3. LC-MS analysis

In separate Eppendorf tubes, plasma and fecal samples were extracted using extraction solvent without added internal standard, as described above, before pooling and using as the diluent for calibration solutions (separate sets for plasma and fecal samples). Calibration standards for Trp, TA, Kyn and the internal standard (3-methyl-2-oxindole) were each prepared individually in methanol. Mixed calibration solutions containing all standards and internal standard (1.25 μM) were subsequently prepared, each at 8 levels, over the following concentration ranges: Trp (0.625–160 μM); TA (3.125–400 nM); Kyn (31.25–4,000 nM), using the pooled extract as a diluent, in order to standardize for ionization suppression. The calibration solutions were prepared by mixing 5 μL of the standard mix solution with 95 μL of plasma extract. Endogenous concentrations of each metabolite were determined by extrapolation to the *y*-axis intercept of the calibration curve (zero added standard).

LC-MS analysis was performed using a Dionex RSLC3000 ultra high-performance liquid chromatograph coupled to a Q-Exactive Plus Orbitrap mass spectrometer (Thermo Fisher Scientific Australia Pty Ltd., Scorseby, VIC, Australia). Samples were analyzed using reverse phase chromatography with a C18 2.1 × 100 mm, 1.8 μm, Zorbax Eclipse Plus and equivalent 5 mm guard column (Agilent Technologies Australia Pty Ltd., Mulgrave, VIC, Australia). A gradient elution of 0.1% formic acid (A) and acetonitrile 0.1% formic acid (B) (linear gradient time-%B: 0 min-0%, 9.5 min-36%, 10.5 min-95%, 12.5 min-95%, 13.0 min-0%, 16 min-0%) was utilized at 40°C. The flow rate was maintained at 300 μL/min. Samples were maintained at 6°C pending injection (10 μL). Mass spectrometry was performed using electrospray ionization in positive ion mode (4 kV, capillary temperature 300°C; sheath gas flow rate 50; auxiliary gas flow rate 20; spare gas 0; probe temp 120°C) cycling between full scan MS at resolution 70,000 and parallel reaction monitoring mode targeting the protonated ions of Trp, Kyn, TA and 3-methyl-2-oxindole (Resolution = 17,500, automatic gain control target 2e5, Max injection time = 100 ms, Isolation window 1.2 m/z and (N)CE/stepped nce: 35). Data analysis was performed using TraceFinder 4.1 software (Thermo Fisher Scientific Inc., Scoresby, VIC, Australia). The concentration of each metabolite was determined by comparison with its corresponding calibration curve and correction for the background level of metabolite introduced from the pooled extract.

### 2.6. Statistical analysis

Datasets including body weight, feed intake, blood and fecal metabolites were tested for normality using the Shapiro–Wilk test and then analyzed using the non-parametric Mann–Whitney U test. Significance differences were reported at *p* < 0.05.

## 3. Results

The median weight of pigs on arrival was 8.0 kg (standard diet) and 6.5 kg (resistant protein diet) and at the time of euthansia were 18.8 kg (standard diet) and 8.0 kg (resistant protein diet) (Table 1). The significantly lower body mass gain of the resistant protein diet animals most likely reflected the significantly lower intake of the feed (Table 1) and its significantly lower protein digestibility, as shown previously (16).

**TABLE 1:** Average body mass and feed intakes of pigs for each group (*n* = 4).

Measure	^14^C-ALA	^14^C-Tyr
Starting weight (kg)	8.0 (7.5, 9.0)	6.5 (6.0, 7.0)
Final weight at day F (kg)	18.8 (15.0, 19.0)	8.0 (8.0, 8.5)
Average feed intake (g/day)	613.1 (555.2, 683.9)	287.8 (280.3, 298.0)
Average energy intake (kJ/day)	8,154 (7,385, 9,095)	3,828 (3,728, 3,963)

Data are resented as median (minimum, maximum). For all measures, the averages between groups were significantly different, *p* < 0.05, by Mann–Whitney U test.

### 3.1. Biodistribution of ^14^C-ALA and metabolites

**FIGURE 3:**
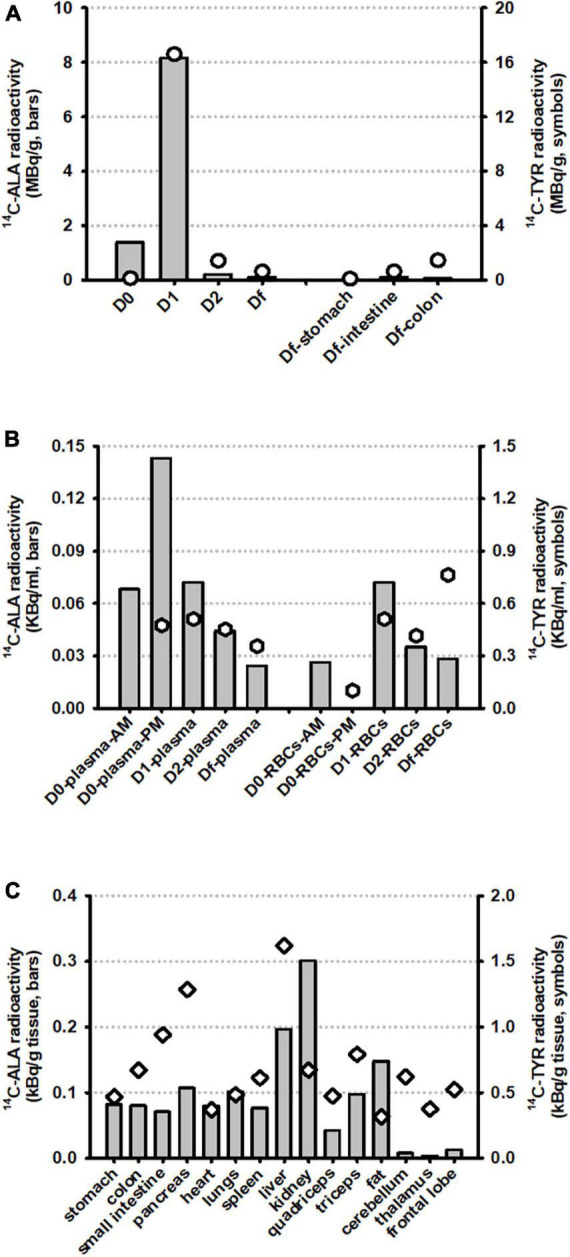
Biodistribution of radioactivity from ^14^C-ALA (*n* = 1) and ^14^C-Tyr (*n* = 1) at a total dose of 20 MBq. The results are presented for **(A)** excreta samples (urine plus feces, taken on days 0, 1, 2, and F) and digesta samples, taken from the stomach, small intestine and colon on day F; **(B)** Blood plasma and blood cells (RBCs) on days 0, 1, 2, and F; and **(C)** Organ and tissue samples taken on day F.

A large proportion of ^14^C-ALA radioactivity (52.4%) was excreted in the urine and feces, peaking on day 1 and declining significantly at day 2 and day F (Figure 3A). Very low levels of radioactivity were detected in digesta from the stomach, small intestine or colon on day F (Figure 3A and Table 2). ^14^C-ALA radioactivity was detected in plasma from day 0 and peaked in both plasma and blood cells on day 1 (Figure 3B). Only 1.45% of the original dose of radioactivity was recovered in the organs (stomach, small intestine, colon, liver, pancreas, kidney, spleen, heart, lungs, and brain) (Table 2). The highest amount of total radioactivity in the organs was detected in the liver (0.62%), followed by the small intestine (0.21%). The highest concentrations of ^14^C-ALA radioactivity were found in the kidney (0.002% dose/g), liver (0.001% dose/g) and fat tissue (0.001% dose/g). The levels of ^14^C-ALA radioactivity were very low in the brain (<0.01%).

**TABLE 2:** Radioactivity measured in blood, excreta, and selected tissues and organs for ^14^C-ALA (*n* = 1) and ^14^C-Tyr (*n* = 1), as concentration and percentage of original dose (20 MBq).

Day	Sample	^14^C-Tyr (kBq/g or kBq/mL)	^14^C-Tyr (% of dose)	^14^C-ALA (kBq/g or kBq/mL)	^14^C-ALA (% of dose)
0	Plasma	0.48	0.85	0.11	0.41
	Blood cells	0.10	0.08	0.03	0.04
	Excreta	0.14	0.19*	1.37	3.95*
1	Plasma	0.51	0.91	0.07	0.28
	Blood cells	0.51	0.39	0.07	0.12
	Excreta	16.58	43.12*	8.14	46.80*
2	Plasma	0.45	0.81	0.04	0.17
	Blood cells	0.42	0.32	0.04	0.06
	Excreta	1.43	3.71*	0.19	1.09*
F	Plasma	0.36	0.64	0.02	0.09
	Blood cells	0.76	0.58	0.03	0.05
	Excreta	0.62	1.62*	0.10	0.57*
	Digesta from stomach	0.10	NA	<0.01	NA
	Digesta from intestine	0.64	NA	0.10	NA
	Digesta from colon	1.46	NA	0.06	NA
	Quadricep	0.47	NA	0.04	NA
	Tricep	0.79	NA	0.10	NA
	Colon	0.67	0.58	0.08	0.17
	Small intestine	0.94	3.08	0.07	0.21
	Stomach	0.47	0.22	0.08	0.08
	Spleen	0.61	0.00	0.08	0.02
	Heart	0.37	0.00	0.08	0.04
	Liver	1.62	2.24	0.20	0.62
	Kidney	0.67	0.16	0.30	0.18
	Lungs	0.48	0.30	0.10	0.11
	Pancreas	1.29	0.14	0.11	0.02
	Subcutaneous adipose tissue	0.32	NA	0.15	NA
	Brain lobes	0.52	0.09	0.01	<0.01
	Brain cerebellum	0.62	0.03	0.01	<0.01
	Brain mid-brain	0.37	0.03	<0.01	<0.01

*Determined from estimated total urine and feces and total blood volume, based on body sizes. Plasma and blood cells were estimated at a ratio of 70:30 of total blood volume. NA, data not available for tissues/samples where total mass could not be measured or estimated.

### 3.2. Biodistribution of ^14^C-Tyr and metabolites

A large proportion of ^14^C-Tyr radioactivity (48.6%) was excreted in the urine and feces, peaking on day 1 and declining significantly at day 2 and day F (Figure 3A). The detection of relatively higher levels of radioactivity in the colon digesta, compared with the stomach and small intestine, on Df supports the idea that the tyrosine was delivered to colon for microbial fermentation, as intended (Figure 3A). Radioactivity was detected in plasma from day 0 to day F, with a peak observed day 1. Radioactivity was also observed in blood cells from day 0 to day F, with a peak on day F (Figure 3B).

Of the original dose, 6.9% was collectively recovered in all the organs sampled (stomach, small intestine, colon, liver, pancreas, kidney, spleen, heart, lungs, and brain) (Table 2). The highest amount of total radioactivity in the organs was detected in the small intestine (3.1%), followed by the liver (2.2%), The highest concentrations of radioactivity were detected in the liver (0.008% dose/g) and pancreas (0.006% dose/g). Radioactivity of 0.007% dose/g was detected in the colon digesta. Significantly, of the initial 20 MBq, 0.14% of the radioactivity was detected in the brain, with the highest quantity detected in the brain lobes (0.088%) and highest concentration detected in the cerebellum (0.003% dose/g). The results support that ^14^C-Tyr or its metabolites are bioavailable to the brain.

### 3.3. Analysis of tryptophan metabolites

Metabolites related to the tryptophan metabolism pathway (Trp, Kyn, TA) were analyzed in plasma and feces. In this context, the samples from the standard diet group act as a control for the resistant protein diet group in examining the effects of reduced protein digestibility and gut microbial fermentation on the metabolites of an aromatic amino acid.

**FIGURE 4:**
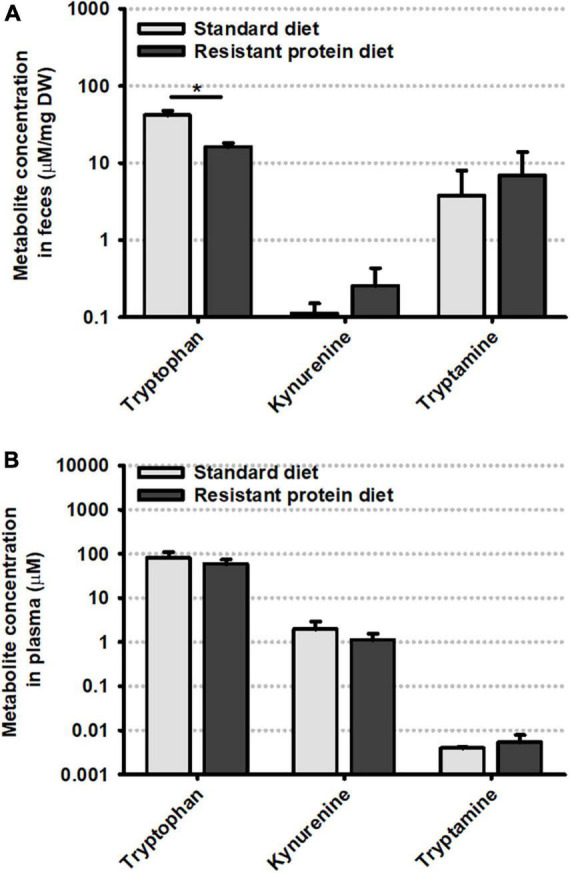
Comparison of levels of tryptophan and its metabolites, kynurenine and tryptamine in **(A)** feces and **(B)** plasma, on day -3. Data represent means and standard deviation (*n* = 3). Statistical comparisons were conducted using the non-parametric Mann–Whitney U test with significant differences shown at *p* < 0.05.

**FIGURE 5:**
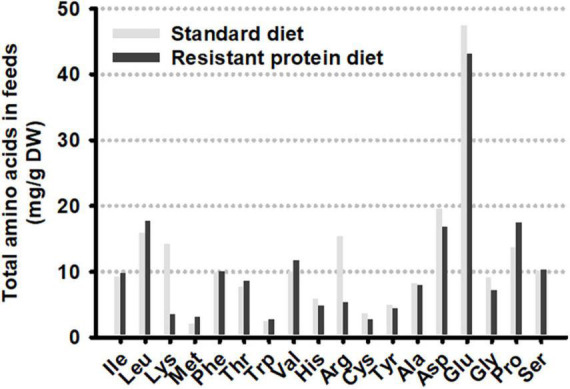
Total amino acid composition of the two formulated diets.

The fecal concentration of tryptophan was greater in animals on the standard diet compared to the resistant protein diet (Figure 4A). Conversely, the average fecal concentration of kynurenine and tryptamine was lower on the standard diet although this failed to reach statistical significance. In contrast, there was no difference in the plasma concentration of these metabolites (Figure 4B). The variations in fecal metabolite concentrations did not appear to be correlated with level of tryptophan in the diet as it was very similar in both the standard and resistant feeds (Figure 5). Amino acid profiles of the feeds from the two diets were similar for the majority of the amino acids with the exception of lysine and arginine which were significantly lower in the resistant protein diet consistent with their susceptibility to modification by the Maillard reaction.

## 4. Discussion

This research investigates the biodistribution of microbially fermented Tyr, and its metabolites, and the concentration of microbially and normally metabolized Trp to contribute to our understanding of the relationship between gut microbiota, fermentation of dietary protein, and brain function. This represents a key step in characterizing the relationship between nutrition and mental health, and understanding the “food-gut-brain axis” (10, 11). This study investigated the biodistribution of ^14^C-labeled Tyr (as a test compound) and ALA (as a model compound), as well as the concentration of Trp, Kyn and TA in plasma and feces. The study was conducted in weanling pigs, in order to maximize the concentration of radioactive metabolites in tissues. The feed characteristics and health impacts of the different feeding treatments were reported previously (16). The focus of the data reported here is to highlight the biodistribution of ^14^C-nutrients and their metabolites, with a particular interest in biodistribution of gut microbial fermented tyrosine and its localization in brain tissues, and to highlight alternations in amino acid metabolism from a high resistant protein diet.

### 4.1. Biodistribution of ^14^C-ALA accompanying a standard diet

Omega-3 fatty acids are involved in many important normal functions in the human body, are linked with the intrinsic nutritional value of fish and also incorporated as ingredients to increase the healthiness of other food products (33). In particular, ALA is an essential fatty acid in the human diet and a biosynthetic precursor for the long-chain eicosapentaenoic acid (EPA) and docosahexaenoic acid (DHA) (27), both of which are important for human health (34–36). In the current study, ALA was chosen as a reference compound whose metabolism and biodistribution is well defined (27), for validating the mapping of biodistribution using the workflow.

The accepted metabolism of ALA in mammals following digestion and absorption in the upper GI tract is transport to liver in chylomicrons, followed by incorporation of the ALA into plasma lipoproteins and distribution and incorporation of the ALA to muscle, adipose, skin and other organs (28). ALA does not accumulate to any extent in either the red blood cells or the brain (37). A major route of disposal of ingested ALA is *via* beta-oxidation to CO_2_, presumably occurring in muscle (28). It has been shown that between 30 and 60% of ^14^C-ALA is expired as CO_2_ within the first 24 h (28, 38). In the liver, ALA is also metabolized to longer chain polyunsaturated fatty acids (PUFA) *via* a sequence of desaturation and chain elongations to yield EPA, docosapentaenoic acid and DHA (39). These longer chain PUFAs are also transported around the body from the liver in plasma lipoproteins. Tissues which accumulate DHA include brain and retina (37, 40). Similar to observations in the present study, studies in suckling rat pups, and guinea pigs following the metabolic fate of orally dosed ^14^C-ALA, have shown significantly higher levels of ^14^C in the liver than the brain (29, 41). Several studies have revealed substantial deposition of ALA in the skin, in rats and guinea pigs (37, 42) and extensive ^14^C-labeling from oral doses of ^14^C-ALA in the skin and fur of guinea pigs (29).

In this study, the remarkable observation of the biodistribution of the orally administered ^14^C-ALA in a pig was that the majority of the label was excreted in feces and urine (46.8% on day 2), with less than 9% recovered in other tissues (Figure 3C and Table 2). The high level of excretion of the ^14^C from ALA in feces and urine has not been reported previously. Excretion of ALA *via* this route has been reported to be less than 1% of the fed ALA (43). Previous studies have provided the oral ^14^C-ALA mixed into a vegetable oil (29, 41), while in the current study, the ^14^C-ALA was mixed with methocel and contained in a capsule. It is possible that the capsule or the methocel or both limited the digestion and absorption of the ^14^C-ALA, by delaying uptake until later in the GI tract after the capsule was fully dissolved.

Despite the high level of excretion of the ^14^C-label from ALA *via* the feces and urine, there was extensive labeling of muscle [estimated at 6.6% of administered dose, based on body composition data (44)], adipose (estimated at 0.7%) and liver (0.6%) with lower levels of uptake of ^14^C-ALA in kidney, lungs, small intestine and colon (between 0.1 and 0.2% of dose), and very low levels of ^14^C detected in brain (<0.002% of dose). The signal of ^14^C-ALA was highest in kidney tissue (0.002% dose/g). The ^14^C labeling of the skin was not collected in this study. This tissue labeling pattern is consistent with studies in rats and guinea pigs (29, 41). The total mass recovery of the ^14^C-ALA was approximately 61% of the dose administered. It is presumed that the remaining 39% of the dose was eliminated *via* metabolism of the ^14^C-ALA to CO_2_. The ^14^C-ALA biodistribution data provided an independent reference nutrient that confirmed expected biodistribution and validated the research methodology.

### 4.2. Biodistribution of ^14^C-Tyr accompanying a resistant protein diet

**FIGURE 6:**
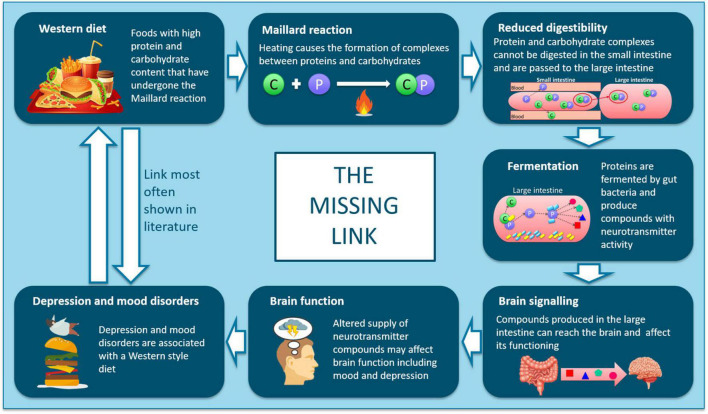
Schematic showing the proposed mechanisms linking intake of ultra-processed, resistant protein-containing foods with outcomes relating to depression and mood disorders.

The link between poor diet (specifically a Western-style diet), gut microbiota and mental health has been previously highlighted (11, 45–47), however, a clear biological pathway for the link between poor diet and poor mental health remains unproven. We hypothesize that high levels of resistant protein may be a key dietary factor that contributes to poor mental health. In relation to characterizing the “missing link” between diet and mental health, we have previously demonstrated the “Maillard reaction” and “reduced digestibility” elements of the pathway (Figure 6) (16). High-heat processing of food, driving Maillard chemistry between proteins and reducing carbohydrates, resulted in dietary proteins that were poorly digestible (resistant proteins) and passed through to the large intestine where they were fermented by the gut microbiota and influenced microbial composition (16). The “fermentation” element has been previously reported, detailing the production of neuroactive and neurotransmitter compounds from the microbial fermentation of amino acids (15, 48, 49). The results from this study demonstrate that tyrosine or tyrosine-derived metabolites, digested by a resistant protein-primed gut microbiota, can access the brain (Figure 3C and Table 2). Therefore, there is potential for these compounds to have neuroactive effects, contributing toward the “brain signaling” element in understanding the link between poor diet and poor mental health.

Tyrosine is an aromatic amino acid and precursor to key neurotransmitters, dopamine, adrenaline and noradrenaline, which have profound effects on mood, reward behavior, wakefulness and motor activity (46). Dietary tyrosine depletion has been implicated in an increased risk of clinical depression (50). When amino acids, like tyrosine, are fermented by gut microbes, they may be converted to potentially toxic compounds, such as ammonia, amines, N-nitroso compounds, phenols, cresols, indoles and hydrogen supplied (18, 51–53), rendering them unavailable as neurotransmitter precursors and mimicking dietary depletion. However, gut microbes also have the potential to convert amino acids to neurotransmitters (e.g., dopamine, serotonin) that function as signaling molecules in the enteric nervous system, with systemic and brain accessibility, known as the “gut-brain axis” (15, 48, 54). This aligns with the growing understanding of regulation of brain functions and mood *via* the gut-brain axis (55). Significantly, this study has demonstrated the bioavailability to brain of gut microbially fermented ^14^C-Tyr and/or its metabolites.

This study also investigated whether feeding a resistant protein diet may alter the concentration of aromatic amino acid and neurotransmitter precursor, Trp, and its metabolites, in plasma and feces. Trp is an essential amino acid that is the precursor to serotonin, a neuromodulator involved in mood, appetite and gastrointestinal function (56). Dietary Trp depletion, resulting in low brain serotonin levels, is associated depressed mood (57). Trp is present in the body in relatively low quantities and its metabolism is vulnerable to changes in physiological status (56) and can be moderated by the gut microbiota (57). The reduction in Trp concentration in the feces observed in the present study may be due to destruction of Trp during the high heat processing of the diet or due to the lower feed intake in the resistant protein diet group, both of which could lead to reduced intake of Trp and the role of either pathway cannot be confirmed. Under normal conditions, 90–95% of ingested Trp is converted to Kyn and enters the kynurenine pathway (57, 58). However, Kyn metabolism can be altered by inflammation and, in turn, changes in Kyn metabolism can influence immune-inflammatory processes (58). In the present study, no differences in Kyn concentration between the resistant protein diet group and standard diet group were detected in the plasma or feces. Given the reduced protein digestibility of the resistant protein diet (16), we had expected to see a reduction in Kyn due to increased microbial fermentation of Trp to produce other metabolites. However, the small sample size presented here suggests that these findings should be interpreted with caution.

Tryptamine (TA) is a microbial fermentation product of Trp, and indicates alterations in Trp metabolism as a result of protein indigestibility and fermentation by the gut microbiota (48, 49). Though no significant differences between the diet groups were identified, the non-significant trend toward elevated TA in the feces and plasma of animals on the resistant protein diet may reflect increased microbial fermentation of Trp into TA from the resistant protein diet compared with the standard diet (15, 16, 18). We have previously reported that the resistant protein diet was accompanied by an altered microbiome composition compared with the standard diet, suggestive of an altered fermentation substrate (16). This finding suggests that some Trp from the resistant protein diet bypassed normal protein digestion and was fermented by the gut microbiota to form TA, which may limit the amount of Trp available to the brain to produce neurotransmitters such as serotonin and dopamine (50, 59, 60). Depletion of amino acids, such as tyrosine and tryptophan, has been linked with neuropsychological changes and increased risk of depression and anxiety in healthy adults (46, 50, 59–62). Meanwhile, overproduction of indole, another Trp metabolite produced by the gut microbiota, was associated with increased anxiety-like behavior in rats (63). This suggests that either the production of toxic metabolites (e.g., indole) and/or the reduced availability of amino acid precursors to the brain may be implicated in neuropsychological conditions (52, 64).

### 4.3. Applications of the proposed workflow

In the absence of radio-labeled nutrient research there are many mechanistic relationships in nutrition and health that remain ambiguous, particularly when activity occurs in tissues and organs that cannot be easily sampled, such as in the brain. Further understanding of how diet increases the risk of poor mental health is required in order to establish ways of reversing this effect (11). These represent worthy research targets for application of the proposed ^14^C workflow (Figure 1). For example, aromatic amino acids, Tyr and Trp, are precursors to neurotransmitters dopamine (Tyr), epinephrine (Tyr), norepinephrine (Tyr) and serotonin (Trp) (46, 65). The digestibility and bioavailability of these amino acids is compromised in resistant proteins (16, 17, 66). This may reduce the supply of neurotransmitter precursors available to the brain and result in the production of other potentially neuroactive or toxic compounds (e.g., indole) that may impact brain function (10, 15, 18, 19, 48, 51). The detection and identification of ^14^C labeled metabolites of amino acids found in the brain would allow further mechanistic work to be done, in established brain cell models, to determine their effects on brain function. This may have important implications for the dietary management of mental health conditions.

The proposed workflow provides a framework for determining the mechanistic functions of bioactive compounds from food by understanding the biodistribution of metabolites within tissues/organs, studying the mechanisms of those metabolites in cell assays, and measuring those same metabolites along with health markers in clinical studies. This allows deep understanding of the bioactivity of key nutrients in the tissues in which they are active. This process cannot be achieved without radio-isotope labeling. Radio-isotope labeling of molecules is a well-established tool used in pre-clinical and other research fields, typically focusing on small molecules with synthetic pathways that permit control of the localization of the isotope atom(s). The use of ^14^C-labeling to trace the metabolic fate of nutrients is also well-established (21–25), but typically focusses only on biodistribution. The proposed workflow for characterizing biodistribution of nutrients and then identifying their functional activity in relevant tissues is relatively new (20). With the costs of radio-labeled compounds and specialized infrastructure being relatively high, the cost-benefit lies in the highly conclusive data and outcomes from well-designed research targets, as demonstrated in the current study. Likewise, by identifying suitable compounds active within a target pathway, it may be possible to study proposed nutritional origins of other conditions, such as epilepsy (67). We are seeking collaboration and partnerships to extend the applications of this workflow.

### 4.4. Strengths and limitations

A strength of the study was the use of the ^14^C-ALA as a “reference” nutrient (given on a standard diet and for standard digestion and metabolism) to confirm the concepts of the workflow and demonstrate biodistribution of a standard nutrient. This validates the findings reported for “test” nutrient, ^14^C-Tyr, encapsulated for targeted delivery to and microbial fermentation in a gut microbiome primed on a resistant protein diet. However, the prohibitive costs of administering ^14^C-labeled nutrients to clinically relevant large animal models (such as the pig) meant the inclusion of controls for ^14^C-Tyr in which the nutrient was delivered following a standard diet (as compared to the resistant protein diet) or delivered in a standard capsule (as compared to an acid-resistant capsule) were not possible. Therefore, the results of this study can only demonstrate the tissue distribution of tyrosine and its metabolites following metabolism by the resistant protein diet-primed gut microbiota, and cannot indicate how this may have changed from tyrosine metabolism on a standard diet. We have previously compared the effects of the standard and resistant protein diets on the microbiome and other health markers in pigs (16). The specificity of the ^14^C radiolabel is an important strength of this research as it allows for accurate measurement of the biodistribution of a compound. However, while a pill popper (to prevent chewing) and acid-resistant capsule (to prevent gastric digestion) were used together to deliver the ^14^C-Tyr to the gut microbiota, there is the possibility that some ^14^C-Tyr was released and absorbed in the small intestine. Therefore the biodistribution of ^14^C radioactivity, without chemical identification of metabolites and without comparison to metabolism following a standard diet, should be interpreted with caution.

### 4.5. Conclusion

Two different ^14^C-labeled nutrients were used to demonstrate a workflow representing a new and powerful research approach to understand *in vivo* metabolism and biodistribution of nutrients. ^14^C-ALA was given as a model nutrient to validate the workflow. Apart from the high loss of ^14^C-ALA radioactivity (∼52%) in urine and feces (likely due to the encapsulation technique), the remaining biodistribution of radioactivity was as expected based on existing literature, with the highest levels in liver, kidney and fat, and lowest levels in brain tissues. ^14^C-Tyr was given as a test nutrient to investigate the biodistribution of metabolites of the amino acid following gut microbial fermentation (as a model of dietary resistant protein digestion and metabolism). There was also a high loss of ^14^C-Tyr radioactivity (∼49%) in urine and feces. The key finding was that ^14^C-Tyr radioactivity was detected in the brain, suggesting that microbially fermented tyrosine or its metabolites may be able to influence brain function. We previously reported that the resistant protein diet produced an altered microbiome (16). Here, the trends toward increased production of tryptamine, a microbial metabolite of tryptophan, reflects the delivery of amino acids to the colon for microbial fermentation.

The research has, for the first time, demonstrated a step-wise pathway between dietary intake, gut microbiota and brain. Building on our previous findings (16), this research suggests that gut microbial fermentation of dietary resistant protein might impact brain function through affecting the supply of amino acid-derived neurotransmitters or altering amino acid metabolism toward production of potentially toxic metabolites and pro-inflammatory processes. These findings add to our understanding of the missing link between diet and mental health. However, further research is required to identify the compounds that gain access to the brain and define their activity on brain function. Followed by clinical research to validate the association of high resistant protein intake and production of amino acid fermentation metabolites with outcomes related to depression and anxiety.

## Data availability statement

The original contributions presented in this study are included in the article/supplementary material, further inquiries can be directed to the corresponding author.

## Ethics statement

The animal study was reviewed and approved by the Monash Animal Research Platform-1 Animal Ethics Committee (Reference: 17533).

## Author contributions

MM: methodology, formal analysis, investigation, writing—original draft, visualization, data curation, project administration, and writing—review and editing. CB and SB: investigation, data curation, and writing—review and editing. MB: conceptualization, funding acquisition, and writing—review and editing. AG: methodology, investigation, and writing—review and editing. BG, PL, and AS: writing—review and editing. LK: investigation, formal analysis, data curation, and writing—review and editing. SS-P: conceptualization, methodology, and writing—review and editing. HW: investigation, data curation, and writing—review and editing. GW: supervision, methodology, and writing—review and editing. LB: conceptualization, methodology, investigation, resources, writing—original draft, visualization, supervision, writing—review and editing, and funding acquisition. All authors contributed to the article and approved the submitted version.

## References

[1] van Kleef E van Trijp HCM Luning P. Functional foods: health claim-food product compatibility and the impact of health claim framing on consumer evaluation. *Appetite.* (2005) 44:299–308. 10.1016/j.appet.2005.01.009 15894404

[2] Srour B Touvier M. Ultra-processed foods and human health: what do we already know and what will further research tell us? *EClinicalMedicine.* (2021) 32:100747. 10.1016/j.eclinm.2021.100747 33644723 PMC7889793

[3] Ratnayake WMN Galli C. Fat and fatty acid terminology, methods of analysis and fat digestion and metabolism: a background review paper. *Ann Nutr Metab.* (2009) 55:8–43. 10.1159/000228994 19752534

[4] Arballo J Amengual J Erdman JW. Lycopene: a critical review of digestion, absorption, metabolism, and excretion. *Antioxidants.* (2021) 10:342. 10.3390/antiox10030342 33668703 PMC7996133

[5] Murray M Dordevic AL Ryan L Bonham MP. An emerging trend in functional foods for the prevention of cardiovascular disease and diabetes: marine algal polyphenols. *Crit Rev Food Sci Nutr.* (2018) 58:1342–58. 10.1080/10408398.2016.1259209 27834493

[6] Berding K Vlckova K Marx W Schellekens H Stanton C Clarke G Diet and the microbiota-gut-brain axis: sowing the seeds of good mental health. *Adv Nutr.* (2021) 12:1239–85. 10.1093/advances/nmaa181 33693453 PMC8321864

[7] Dash S Clarke G Berk M Jacka FN. The gut microbiome and diet in psychiatry: focus on depression. *Curr Opin Psychiatry.* (2015) 28:1–6. 10.1097/YCO.0000000000000117 25415497

[8] Järbrink-Sehgal EJ Andreasson A. The gut microbiota and mental health in adults. *Curr Opin Neurobiol.* (2020) 62:102–14. 10.1016/j.conb.2020.01.016 32163822

[9] Grosso G. Nutritional psychiatry: how diet affects brain through gut microbiota. *Nutrients.* (2021) 13:1282. 10.3390/nu13041282 33919680 PMC8070365

[10] Ceppa F Mancini A Tuohy K. Current evidence linking diet to gut microbiota and brain development and function. *Int J Food Sci Nutr.* (2018) 70:1–19. 10.1080/09637486.2018.1462309 29671359

[11] Jacka FN. Nutritional psychiatry: where to next? *EBioMedicine.* (2017) 17:24–9. 10.1016/j.ebiom.2017.02.020 28242200 PMC5360575

[12] Dodd D Spitzer MH Van Treuren W Merrill BD Hryckowian AJ Higginbottom SK A gut bacterial pathway metabolizes aromatic amino acids into nine circulating metabolites. *Nature.* (2017) 551:648–52. 10.1038/nature24661 29168502 PMC5850949

[13] Alam R Abdolmaleky HM Zhou JR. Microbiome, inflammation, epigenetic alterations, and mental diseases. *Am J Med Genet B Neuropsychiatr Genet.* (2017) 174:651–60. 10.1002/ajmg.b.32567 28691768 PMC9586840

[14] Neis E Dejong C Rensen S. The role of microbial amino acid metabolism in host metabolism. *Nutrients.* (2015) 7:2930–46. 10.3390/nu7042930 25894657 PMC4425181

[15] Portune KJ Beaumont M Davila A-M Tomé D Blachier F Sanz Y. Gut microbiota role in dietary protein metabolism and health-related outcomes: the two sides of the coin. *Trends Food Sci Technol.* (2016) 57:213–32. 10.1016/j.tifs.2016.08.011

[16] Murray M Coughlan MT Gibbon A Kumar V Marques FZ Selby-Pham S Reduced growth, altered gut microbiome and metabolite profile, and increased chronic kidney disease risk in young pigs consuming a diet containing highly resistant protein. *Front Nutr.* (2022) 9:816749. 10.3389/fnut.2022.816749 35399679 PMC8988180

[17] ALjahdali N Carbonero F. Impact of Maillard reaction products on nutrition and health: current knowledge and need to understand their fate in the human digestive system. *Crit Rev Food Sci Nutr.* (2019) 59:474–87. 10.1080/10408398.2017.1378865 28901784

[18] Macfarlane GT Macfarlane S. Bacteria, colonic fermentation, and gastrointestinal health. *J AOAC Int.* (2012) 95:50–60. 10.5740/jaoacint.SGE_Macfarlane 22468341

[19] Lin R Liu W Piao M Zhu H. A review of the relationship between the gut microbiota and amino acid metabolism. *Amino Acids.* (2017) 49:2083–90. 10.1007/s00726-017-2493-3 28932911

[20] Buecking M Oeckener B Haynes JM Leitner P Murray M Williamson G Research tool helps validate efficacy of functional foods. *Food Technol.* (2019) 73:44–8.

[21] Odle J Benevenga NJ Crenshaw TD. Evaluation of [1-14C]-medium-chain fatty acid oxidation by neonatal piglets using continuous-infusion radiotracer kinetic methodology. *J Nutr.* (1992) 122:2183–9. 10.1093/jn/122.11.2183 1432258

[22] Heo KN Lin X Han IK Odle J. Medium-chain fatty acids but not L-carnitine accelerate the kinetics of [14C]triacylglycerol utilization by colostrum-deprived newborn pigs. *J Nutr.* (2002) 132:1989–94. 10.1093/jn/132.7.1989 12097681

[23] Schweigert FJ Rosival I Rambeck WA Gropp J. Plasma transport and tissue distribution of [14C] beta-carotene and [3H]retinol administered orally to pigs. *Int J Vitam Nutr Res.* (1995) 65:95–100. 7591538

[24] Wang KS Kearns GL Mock DM. The clearance and metabolism of biotin administered intravenously to pigs in tracer and physiologic amounts is much more rapid than previously appreciated. *J Nutr.* (2001) 131:1271–8. 10.1093/jn/131.4.1271 11285337

[25] Prelusky DB Miller JD Trenholm HL. Disposition of 14C-derived residues in tissues of pigs fed radiolabelled fumonisin B1. *Food Addit Contam.* (1996) 13:155–62. 10.1080/02652039609374393 9064240

[26] Sciascia Q Daş G Metges CC. Review: the pig as a model for humans: effects of nutritional factors on intestinal function and health. *J Anim Sci.* (2016) 94:441–52. 10.2527/jas.2015-9788 32704858

[27] Burdge GC. Metabolism of α-linolenic acid in humans. *Prostaglandins Leukot Essent Fatty Acids.* (2006) 75:161–8. 10.1016/j.plefa.2006.05.013 16828546

[28] Sinclair AJ Attar-Bashi NM Li D. What is the role of alpha-linolenic acid for mammals? *Lipids.* (2002) 37:1113–23. 10.1007/s11745-002-1008-x 12617463

[29] Fu Z Attar-Bashi NM Sinclair AJ. 1-C-14-Linoleic acid distribution in various tissue lipids of guinea pigs following an oral dose. *Lipids.* (2001) 36:255–60. 10.1007/s11745-001-0715-7 11337980

[30] Kilkenny C Browne WJ Cuthill IC Emerson M Altman DG. Improving bioscience research reporting: the ARRIVE guidelines for reporting animal research. *PLoS Biol.* (2010) 8:e1000412. 10.1371/journal.pbio.1000412 20613859 PMC2893951

[31] Boyd BJ Kaminskas LM Karellas P Krippner G Lessene R Porter CJ. Cationic poly-L-lysine dendrimers: pharmacokinetics, biodistribution, and evidence for metabolism and bioresorption after intravenous administration to rats. *Mol Pharm.* (2006) 3:614–27. 10.1021/mp060032e 17009860

[32] Zhu W Stevens AP Dettmer K Gottfried E Hoves S Kreutz M Quantitative profiling of tryptophan metabolites in serum, urine, and cell culture supernatants by liquid chromatography-tandem mass spectrometry. *Anal Bioanal Chem.* (2011) 401:3249–61. 10.1007/s00216-011-5436-y 21983980

[33] Kaur N Chugh V Gupta AK. Essential fatty acids as functional components of foods- a review. *J Food Sci Technol.* (2014) 51:2289–303. 10.1007/s13197-012-0677-0 25328170 PMC4190204

[34] Sinn N Milte CM Street SJ Buckley JD Coates AM Petkov J Effects of n-3 fatty acids, EPA v. DHA, on depressive symptoms, quality of life, memory and executive function in older adults with mild cognitive impairment: a 6-month randomised controlled trial. *Br J Nutr.* (2012) 107:1682–93. 10.1017/S0007114511004788 21929835

[35] Mozaffarian D Wu JHY. (n-3) Fatty acids and cardiovascular health: are effects of EPA and DHA shared or complementary? *J Nutr.* (2012) 142:614S–25S. 10.3945/jn.111.149633 22279134 PMC3278271

[36] Sinclair AJ. Docosahexaenoic acid and the brain- what is its role? *Asia Pac J Clin Nutr.* (2019) 28:675–88. 31826363 10.6133/apjcn.201912_28(4).0002

[37] Salem NM Lin YH Moriguchi T Lim SY Salem N Hibbeln JR. Distribution of omega-6 and omega-3 polyunsaturated fatty acids in the whole rat body and 25 compartments. *Prostaglandins Leukot Essent Fatty Acids.* (2015) 100:13–20. 10.1016/j.plefa.2015.06.002 26120061 PMC4555191

[38] Pan DA Storlien LH. Dietary-lipid profile is a determinant of tissue phospholipid fatty-acid composition and rate of weight-gain in rats. *J Nutr.* (1993) 123:512–9. 10.1093/jn/123.3.512 8463854

[39] Domenichiello AF Kitson AP Bazinet RP. Is docosahexaenoic acid synthesis from a-linolenic acid sufficient to supply the adult brain? *Prog Lipid Res.* (2015) 59:54–66. 10.1016/j.plipres.2015.04.002 25920364

[40] Abedin L Lien EL Vingrys AJ Sinclair AJ. The effects of dietary alpha-linolenic acid compared with docosahexaenoic acid on brain, retina, liver, and heart in the guinea pig. *Lipids.* (1999) 34:475–82. 10.1007/s11745-999-0387-3 10380119

[41] Sinclair AJ. Incorporation of radioactive polyunsaturated fatty-acids into liver and brain of developing rat. *Lipids.* (1975) 10:175–84. 10.1007/BF02534156 1128172

[42] Fu Z Sinclair AJ. Increased alpha-linolenic acid intake increases tissue alpha-linolenic acid content and apparent oxidation with little effect on tissue docosahexaenoic acid in the guinea pig. *Lipids.* (2000) 35:395–400. 10.1007/s11745-000-537-7 10858024

[43] Poumes-Ballihaut C Langelier B Houlier F Alessandri JM Durand G Latge C Comparative bioavailability of dietary alpha-linolenic and docosahexaenoic acids in the growing rat. *Lipids.* (2001) 36:793–800. 10.1007/s11745-001-0786-5 11592729

[44] Landgraf S Susenbeth A Knap PW Looft H Plastow GS Kalm E Developments of carcass cuts, organs, body tissues and chemical body composition during growth of pigs. *Anim Sci.* (2006) 82:889–99. 10.1017/ASC2006097

[45] Ekmekcioglu C. Are proinflammatory cytokines involved in an increased risk for depression by unhealthy diets? *Med Hypotheses.* (2012) 78:337–40. 10.1016/j.mehy.2011.11.015 22153575

[46] Lang UE Beglinger C Schweinfurth N Walter M Borgwardt S. Nutritional aspects of depression. *Cell Physiol Biochem.* (2015) 37:1029–43. 10.1159/000430229 26402520

[47] Marx W Moseley G Berk M Jacka F. Nutritional psychiatry: the present state of the evidence. *Proc Nutr Soc.* (2017) 76:427–36. 10.1017/S0029665117002026 28942748

[48] Peixin F Linsen L Arash R Shabnam E Dongsheng C Xi M. Metabolites of dietary protein and peptides by intestinal microbes and their impacts on gut. *Curr Protein Pept Sci.* (2015) 16:646–54. 10.2174/1389203716666150630133657 26122784

[49] Williams B Van Benschoten A Cimermancic P Donia M Zimmermann M Taketani M Discovery and characterization of gut microbiota decarboxylases that can produce the neurotransmitter tryptamine. *Cell Host Microbe.* (2014) 16:495–503. 10.1016/j.chom.2014.09.001 25263219 PMC4260654

[50] McLean A Rubinsztein JS Robbins TW Sahakian BJ. The effects of tyrosine depletion in normal healthy volunteers: implications for unipolar depression. *Psychopharmacology.* (2004) 171:286–97. 10.1007/s00213-003-1586-8 12955284

[51] Yao CK Muir JG Gibson PR. Review article: insights into colonic protein fermentation, its modulation and potential health implications. *Aliment Pharmacol Ther.* (2016) 43:181–96. 10.1111/apt.13456 26527169

[52] Zhang LS Davies SS. Microbial metabolism of dietary components to bioactive metabolites: opportunities for new therapeutic interventions. *Genome Med.* (2016) 8:46–46. 10.1186/s13073-016-0296-x 27102537 PMC4840492

[53] Windey K De Preter V Verbeke K. Relevance of protein fermentation to gut health. *Mol Nutr Food Res.* (2012) 56:184–96. 10.1002/mnfr.201100542 22121108

[54] Galland L. The gut microbiome and the brain. *J Med Food.* (2014) 17:1261–72. 10.1089/jmf.2014.7000 25402818 PMC4259177

[55] Foster JA McVey Neufeld K-A. Gut–brain axis: how the microbiome influences anxiety and depression. *Trends Neurosci.* (2013) 36:305–12. 10.1016/j.tins.2013.01.005 23384445

[56] Le Floc’h N Otten W Merlot E. Tryptophan metabolism, from nutrition to potential therapeutic applications. *Amino Acids.* (2011) 41:1195–205. 10.1007/s00726-010-0752-7 20872026

[57] Jenkins TA Nguyen JCD Polglaze KE Bertrand PP. Influence of tryptophan and serotonin on mood and cognition with a possible role of the gut-brain axis. *Nutrients.* (2016) 8:56. 10.3390/nu8010056 26805875 PMC4728667

[58] Muneer A. Kynurenine pathway of tryptophan metabolism in neuropsychiatric disorders: pathophysiologic and therapeutic considerations. *Clin Psychopharmacol Neurosci.* (2020) 18:507–26. 10.9758/cpn.2020.18.4.507 33124585 PMC7609208

[59] Frey A Lampert A Dietz K Striebich S Locher C Fedorenko O Thyrotropin serum concentrations in healthy volunteers are associated with depression-related personality traits. *Neuropsychobiology.* (2008) 56:123–6. 10.1159/000112954 18182832

[60] Suga H Asakura K Kobayashi S Nojima M Sasaki S. Association between habitual tryptophan intake and depressive symptoms in young and middle-aged women. *J Affect Disord.* (2018) 231:44–50. 10.1016/j.jad.2018.01.029 29438897

[61] Robinson OJ Overstreet C Allen PS Pine DS Grillon C. Acute tryptophan depletion increases translational indices of anxiety but not fear: serotonergic modulation of the bed nucleus of the stria terminalis? *Neuropsychopharmacology.* (2012) 37:1963–71. 10.1038/npp.2012.43 22491355 PMC3376328

[62] Young SN. The effect of raising and lowering tryptophan levels on human mood and social behaviour. *Philos Trans R Soc Lond B Biol Sci.* (2013) 368:20110375. 10.1098/rstb.2011.0375 23440461 PMC3638380

[63] Jaglin M Rhimi M Philippe C Pons N Bruneau A Goustard B Indole, a signaling molecule produced by the gut microbiota, negatively impacts emotional behaviors in rats. *Front Neurosci.* (2018) 12:216. 10.3389/fnins.2018.00216 29686603 PMC5900047

[64] Hsiao EY McBride SW Hsien S Sharon G Hyde ER McCue T Microbiota modulate behavioral and physiological abnormalities associated with neurodevelopmental disorders. *Cell.* (2013) 155:1451–63. 10.1016/j.cell.2013.11.024 24315484 PMC3897394

[65] Rajagopal S Sangam SR Singh S Joginapally VR. Modulatory effects of dietary amino acids on neurodegenerative diseases. *Adv Neurobiol.* (2016) 12:401–14. 10.1007/978-3-319-28383-8_22 27651266

[66] Seiquer I Rubio LA Peinado MJ Delgado-Andrade C Navarro MP. Maillard reaction products modulate gut microbiota composition in adolescents. *Mol Nutr Food Res.* (2014) 58:1552–60. 10.1002/mnfr.201300847 24867162

[67] Fusco F Perottoni S Giordano C Riva A Iannone LF De Caro C The microbiota-gut-brain axis and epilepsy from a multidisciplinary perspective: clinical evidence and technological solutions for improvement of in vitro preclinical models. *Bioeng Transl Med.* (2022) 7:e10296. 10.1002/btm2.10296 35600638 PMC9115712

